# Hepatitis B Surface Antigen Activates Unfolded Protein Response in Forming Ground Glass Hepatocytes of Chronic Hepatitis B

**DOI:** 10.3390/v11040386

**Published:** 2019-04-25

**Authors:** Yao Li, Yuchen Xia, Xiaoming Cheng, David E. Kleiner, Stephen M. Hewitt, Julia Sproch, Tong Li, Hui Zhuang, T. Jake Liang

**Affiliations:** 1 Department of Microbiology and Infectious Disease Center, School of Basic Medical Sciences, Peking University Health Science Center, Beijing 100191, China; liyao861224@163.com (Y.L.); tongli08@vip.sina.com (T.L.); 2 Liver Diseases Branch, National Institute of Diabetes and Digestive and Kidney Diseases, National Institutes of Health, Bethesda, MD 20892, USA; yuchen.xia@nih.gov (Y.X.); xiaoming.cheng@nih.gov (X.C.); julia.sproch@nih.gov (J.S.); 3 Laboratory of Pathology, National Cancer Institute, National Institutes of Health, Bethesda, MD 20892, USA; kleinerd@mail.nih.gov (D.E.K.); hewitts@mail.nih.gov (S.M.H.)

**Keywords:** liver disease, chronic hepatitis B, endoplasmic reticulum stress, ground glass hepatocyte, apoptosis, hepatocellular carcinoma

## Abstract

Ground glass hepatocytes (GGHs), a histological hallmark of chronic hepatitis B virus (HBV) infection, contain excessive hepatitis surface antigen (HBsAg) in the endoplasmic reticulum (ER), which is linked to unfolded protein response (UPR). The mechanism by which HBV activates UPR has not been fully defined. To investigate this, HepG2-NTCP cells and primary human hepatocytes (PHHs) were either infected with HBV or transduced with adenoviral vectors expressing replication-competent HBV genome or individual HBV genes. UPR markers were evaluated by qPCR, Western blotting, and immunofluorescence. Apoptosis and cell viability were measured by Caspase-3/7 and ATPlite assay respectively. We found that UPR markers were induced by the overexpression of HBsAg in HepG2-NTCP cells and PHHs. Elevation of UPR-induced genes showed a dose-dependent correlation with HBsAg levels. In HBV-infected livers, GGHs also demonstrated excessive accumulation of HBsAg associated with increased BIP/GRP78 staining, a marker of UPR. Prolonged activation of UPR by HBsAg overexpression induced signs of apoptosis. Overexpression of HBsAg can induce ER stress through protein kinase RNA-like endoplasmic reticulum kinase (PERK) pathway in vitro, and may be linked to the appearance of GGHs. The activation of UPR by HBsAg may sensitize hepatocytes to cell death and result in possible subsequent cellular changes leading to a premalignant phenotype.

## 1. Introduction

Ground glass hepatocytes (GGHs) are a pathological hallmark of chronic hepatitis B. Under light microscopy, the GGH is characterized by a uniformly dull and glassy appearance of the cytoplasm caused by an overabundance of surface antigens (HBsAg) in the endoplasmic reticulum (ER) [1,2]. GGHs have been reported to have a close relationship with different stages of chronic HBV infection [3,4]. Two main types of GGHs have been previously recognized. Type I GGHs are typically scattered individually, harbor HBsAg in the cytoplasm, and tend to present at the early carrier stage. Type II appear in clusters, with the expression of HBsAg typically occurring at the advanced stages, and are frequently associated with cirrhosis or hepatocellular carcinoma (HCC) [2,4,5]. However, the pathological role of HBV in GGHs foundation remains to be fully elucidated.

HBV is a partially double-stranded DNA virus containing four overlapping open reading frames (ORFs) including preS/S, preCore/Core, Pol and X, coding for HBsAg, hepatitis e antigen (HBeAg), hepatitis core antigen (HBcAg), X protein and HBV DNA polymerase, respectively [6,7,8]. HBsAg consists of three types: small (S), middle (M), and large (L) surface proteins. These proteins are synthesized from three in-frame start codons and share the same ORF: in particular, the S, M and LHBsAg are generated by S, preS2+S and preS1+preS2+S, respectively. In the HBV life cycle, secretory proteins such as HBsAg and HBeAg are folded and assembled in the ER of the hepatocyte [9]. The ER is a dynamic organelle that orchestrates protein synthesis, folding, and transport in eukaryotic cells. Under pathological conditions, such as the overexpression of mutant proteins and/or the overburden of secretory protein synthesis, unfolded or misfolded proteins can accumulate in the ER lumen. This is known as ER stress, which further activates unfolded protein response (UPR) [10]. UPR is a cellular mechanism that maintains protein stability by monitoring protein-folding and conducting communication among different compartments of the cell. Three main UPR pathways have been identified, including activating transcription factor 6 (ATF6), inositol-requiring enzyme 1α (IRE1α), and protein kinase RNA (PKR)-like ER-localized kinase (PERK) [10,11]. Under physiological conditions, transducers ATF6, IRE1α, and PERK bind with an ER stress sensor named binding immunoglobulin protein (BIP). BIP is also known as glucose-regulated protein 78 (GRP78) and is a member of the heat shock protein 70 family. Once ER stress occurs, the three transducers detach from BIP/GRP78 and trigger downstream UPR signals [12]. Several studies reported that mutated HBsAg can cause ER stress and induce UPR in vitro and in vivo [13,14,15]. Overexpression of LHBsAg results in a blockage of HBsAg secretion, consequently leading to an activation of ER stress [13]. In addition, two types of PreS mutants have been reported to accumulate in the ER and activate ER stress that may induce DNA damage and genomic instability [14,15]. Transgenic mice expressing a mutant protein with PreS2 deletion demonstrate ER stress by activating the ATF6 pathway of UPR [16]. 

The aim of our work is to investigate any additional mechanisms by which HBV infection causes ER stress and induces UPR and to understand the pathogenic connection between UPR and the appearance of GGHs in HBV infection. In this study, we showed that a high expression of small HBsAg (SHBsAg) can lead to UPR and apoptosis in cell culture, and characterize GGHs as HBV-infected hepatocytes with high levels of HBsAg as well as markers of UPR.

## 2. Materials and Methods

### 2.1. Recombinant Adenoviruses Production

Recombinant adenovirus (AdV) were generated by inserting 1.3× full-length HBV genome, or genes encoding SHBsAg, HBeAg, HBcAg and HBx, and they have been used extensively in the literature for expressing these individual HBV gene products [17]. The AdVs expressing green fluorescent protein (GFP) was used to monitor transduction efficiency. Recombinant AdVs were produced and titrated according to methods described previously [18]. 

### 2.2. Cell Culture and AdVs Transduction

Human kidney cells 293 (ATCC® CRL-1573) were cultured in high glucose Dulbecco’s Modified Eagles Medium (DMEM, Thermo Fisher Scientific, Waltham, MA, USA) supplemented with 10% fetal bovine serum (FBS, Thermo Fisher Scientific, Waltham, MA, USA). HepG2-NTCP cells (kindly provided by Prof. Ulrike Protzer) were cultured in DMEM with 10% FBS as well. Cryopreserved primary human hepatocytes (PHHs) obtained from BD Biosciences (Woburn, MA, USA) were cultured in William’s E Medium (WEM, Thermo Fisher Scientific, Waltham, MA, USA) containing 10% FBS, 1% Penicillin/Streptomycin, 0.17 μM of human insulin (Sigma Aldrich, St. Louis, MO, USA), 10 μM of hydrocortisone 21-hemisuccinate (Sigma Aldrich, St. Louis, MO, USA), and 1.8% DMSO. HepG2-NTCP cells and PHHs were transduced by AdVs at a multiplicity of infection (MOI) of 1. The eBioscience™ protein transport inhibitor cocktail (PTI, Thermo Fisher Scientific, Waltham, MA, USA) and Thapsigargin (Tg, Sigma-Aldrich, St. Louis, MO, USA) were used as positive controls for induction of ER stress and apoptosis, respectively.

### 2.3. HBV Infection

HBV (genotype D, subtype ayw) derived from HepDE19 cells (kindly provided by Prof. Jutao Guo [19]) was concentrated using centrifugal filter devices (Centricon Plus-70, Biomax 100.000, Millipore Corp., Bedford, MA, USA) and titered by HBV-DNA qPCR [20]. For infection, inoculation of cells was performed with MOI of 300 in WEM containing 5% PEG8000 (Sigma Aldrich, St. Louis, MO, USA) for 16 h. After incubation, cells were washed with PBS three times and cultured in WEM as described before [21].

### 2.4. RNA Preparation and Quantitative Real-Time PCRs

Total RNA from HepG2-NTCP cells and PHHs was extracted using Isolate II RNA mini kit (Bioline, Taunton, MA, USA). cDNA was then generated with the cDNA synthesis kit (Thermo Fisher Scientific, Waltham, MA, USA). mRNAs of target UPR markers including BIP/GRP78, ATF4, CHOP, GADD34 and the reference gene TBP were quantified using SYBR green chemistry with LightCycler^®^ 480 System II (Roche Diagnostics Corporation, Indianapolis, IN, USA). The TBP mRNA value was used to normalize the levels of other mRNAs. The primer sequences of these ER stress markers are described in Supplementary Table S1.

### 2.5. Western Blot Assay

Proteins from the cell lysate of AdVs-transduced HepG2-NTCP cells and positive control were extracted in RIPA buffer (Sigma-Aldrich, St. Louis, MO, USA). Mixed with SDS loading buffer (Aviva System Biology Corporation, San Diego, CA, USA), proteins were subjected to Nupage^TM^ 4–12% Bis-Tris gel (Thermo Fisher Scientific, Waltham, MA, USA) and transferred to a nitrocellulose membrane (iBlot^TM^ 2 transfer stacks, Thermo Fisher Scientific, Waltham, MA, USA) with iBlot^TM^ 2 gel transfer device. Antibodies against BIP/GRP78 (Cell Signaling Technology, Danvers, MA, USA), HBsAg (Creative-Diagnostics, Shirley, NY, USA) and GAPDH (Santa Cruz, CA, USA) were used for Western blot; detailed information of antibodies and their usage in various assays is listed in Supplementary Table S2. Membranes were probed with primary antibodies in Odyssey^®^ blocking buffer (LI-COR Biosciences, Lincoln, NE, USA) overnight at 4 °C. After PBS washing three times, membranes were further probed with Goat anti-Mouse/Rabbit IgG (H+L) Superclonal™ Secondary Antibody (1:1000, Thermo Fisher Scientific, Waltham, MA, USA) in blocking buffer for one hour at room temperature. Supersignal^TM^ West Pico Chemiluminescent substrates (Thermo Fisher Scientific, Waltham, MA, USA) were used for chemiluminescence development.

### 2.6. Paraffin-Embedding of Cells and Liver Tissues

HepG2-NTCP cells collected from AdVs transduction or PTI treatment (1×, 6 h) were fixed overnight in 10% neutral buffered formalin. The pelleted cells were then embedded in an agarose plug, subjected to paraffin impregnation, and sectioned on to positively charged slides. Seven HBV-infected and five normal liver tissues (formalin-fixed and paraffin-embedded) were obtained from the laboratory of pathology in NIH. All subjects provided informed consent for inclusion before they participated in the study. The study was conducted in accordance with the Declaration of Helsinki, and the protocol was approved by the Ethics Committee of OSHR Exemption 18-NIDDK-00430.

### 2.7. Immunofluorescent Staining for Cells and Liver Samples

Paraffin-embedded slides were de-paraffinized with Xylene (Sigma-Aldrich, St. Louis, MO, USA), and treated with 100%, 70%, 50% alcohol and distilled water for five minutes each gradually for rehydration. Antigen retrieval was mediated with 1× citrate buffer (K•D Medical, Columbia, MD, USA) with 0.04% tween20 (Sigma-Aldrich, St. Louis, MO, USA) for 20 min at 95 °C prior to immunostaining. Slides were incubated with the blocking solution in PBS containing 10% goat serum (Cell Signaling Technology, Danvers, MA, USA) for one hour. Subsequently, slides were hybridized overnight in 4 °C with primary antibody against BIP/GRP78 (Cell Signaling Technology, Danvers, MA, USA) and HBsAg (Creative-Diagnostics, Shirley, NY, USA) in PBS with 10% goat serum (Cell Signaling Technology, Danvers, MA, USA), and then incubated with AlexaFluor 488 and 568-conjugated secondary antibodies from Life Technologies. Nuclei were counterstained with Hoechst 33,342 (Thermo Fisher Scientific, Waltham, MA, USA) at 1:5000 in PBS. Each step was followed by three washes with PBS. The fluorescence was visualized with an Axio Observer Z1 microscope equipped with a Zeiss LSM 5 Live DuoScan System under an oil-immersion ×40 objective lens (Carl Zeiss, Jena, Germany). Images were acquired using ZEN 2012 software and analyzed with ImageJ software v1.51s. Use of human tissues were carried out following the rules of Declaration of Helsinki of 1975, revised in 2013. The archival liver biopsy samples were obtained from the Laboratory of Pathology of NCI, NIH in an anonymized fashion and their use approved by the NIH Office of Human Subjects Research Protection (Exemption Form 18-NIDDK-00430) and NIH IRB protocol 91-NIDDK-0214.

HepG2-NTCP cells were seeded and cultured on Lab-Tek II borosilicate chamber coverslips (Nunc, Thermo Fisher Scientific, Waltham, MA, USA). Five days after AdVs transduction or treatment with Tg (1 μM, 24 h), cells were fixed with 4% paraformaldehyde (Sigma-Aldrich, St. Louis, MO, USA) for ten minutes, and permeabilized in 0.5% Triton X-100 (Sigma-Aldrich, St. Louis, MO, USA) for ten minutes. The following steps such as blocking and staining were described for immunofluorescence above. Confocal laser scanning microscopic analysis was performed under oil-immersion ×63 objective lens as described previously [22].

### 2.8. Caspase-3/7 Assay and Cell Viability Assay

HepG2-NTCP cells plated in 96-well microplates (1 × 10^5^ cells/well) were transduced with AdVs (MOI of 1) or treated with Tg for 24 h. Five days after transduction, caspase activity was assessed using Caspase-Glo^®^ 3/7 system (Promega, Madison, WI, USA). The caspase-induced chemiluminescence was determined after one-hour incubation using the POLARstar Omega multi-detection microplate reader (BMG LABTECH, Ortenberg, Germany). To assay cell viability, ATP assay solution (AAT Bioquest^®^, Inc., CA, USA) was used. Ten minutes after incubation, luminescence intensity in each well was measured with the same microplate reader.

### 2.9. Statistical Analysis

All results were confirmed by three independent experiments. All data were analyzed with GraphPad Prism5 software (GraphPad Software Inc., La Jolla, CA, USA). Results were described by mean ± standard error of mean (SEM). The error bar means SEM. The Student’s unpaired two-tailed *t* test, Chi-square test or Fisher’s exact test were used for continuous and categorical data analysis, respectively. Spearman rank sum test was applied for correlation statistical analysis. *p* value < 0.05 was considered statistically significant. *p < 0.05, **p < 0.01, ***p < 0.001. 

## 3. Results

### 3.1. Activation of PERK Pathway by Overexpression of HBsAg

PERK is one of the UPR signalling pathways. When ER stress occurs, the transducer separates from BIP/GRP78. This is followed by auto-phosphorylation, leading to the activation of eukaryotic translation-initiation factor α (eIF2α), and the translational suppression of new protein synthesis. ATF4, the other UPR signaling molecule, also separates from BIP/GRP78 and translocates to the nucleus, activating downstream UPR target genes such as the ATF4 gene itself and BIP/GRP78. Other UPR target genes include CCAAT-enhancer-binding protein homologous protein (CHOP), which results in an activation of apoptosis and an induction of growth arrest, and DNA damage 34 (GADD34), which restores mRNA translation [23].

**Figure 1:**
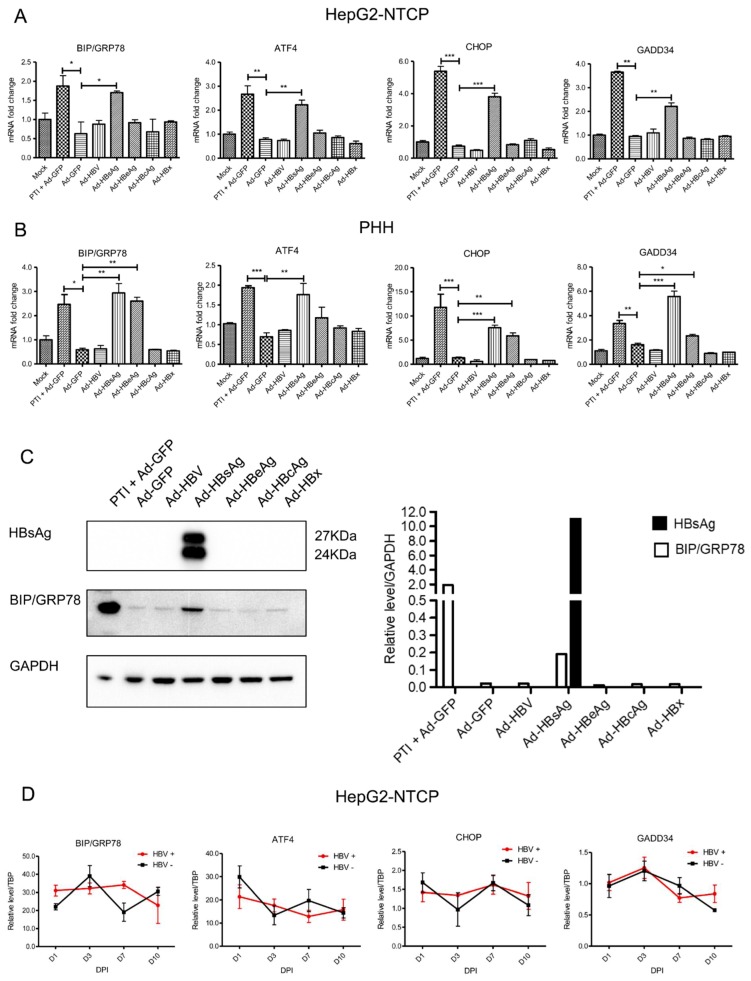
Activation of PERK pathway genes by HBsAg overexpression. (**A**) HepG2-NTCP cells and (**B**) primary human hepatocyte cells were transduced with recombinant AdVs in triplicate (MOI of 1). Protein transportation inhibitor (PTI) was used as a positive control. mRNAs of PERK genes were determined by RT-qPCR three days after AdVs transduction. Relative mRNA expression was normalized to housekeeping gene TBP and mock control. The Student’s unpaired two-tailed *t* test was applied for data analysis. *p < 0.05, **p < 0.01, ***p < 0.001. (**C**) Expression of HBsAg and BIP/GRP78 were determined by Western blotting in AdVs-transduced HepG2-NTCP cells. (**D**) HepG2-NTCP cells were infected with infectious HBV (MOI of 300). UPR markers were analyzed at different days post infection (DPI). Relative level to reference TATA-binding protein (TBP) was calculated. All results were confirmed by three independent experiments.

To determine whether the PERK pathway was affected by HBV and/or HBV proteins, HepG2-NTCP cells and PHHs were transduced with AdVs expressing 1.3× full-length HBV genome or various HBV genes, and PERK pathway genes were evaluated with qPCR. PERK markers, including BIP/GRP78, ATF4, CHOP and GADD34, were measured after AdVs transduction or HBV infection. Because protein transportation inhibitor (PTI) is known to induce ER stress, it was used as a positive control. HBV and HBV proteins were successfully expressed by recombinant AdVs in HepG2-NTCP cells (Supplementary Figure S1). Compared to the negative control (Ad-GFP), mRNA levels of BIP/GRP78, ATF4, CHOP and GADD34 were increased more than two-fold in Ad-HBsAg transduced cells in both HepG2-NTCP cells and PHHs (Figure 1A,B). Interestingly, HBeAg overexpression in PHHs also induced these UPR markers, though to a lesser extent than HBsAg overexpression. This difference suggests that PHHs may be more sensitive to UPR. To further confirm the data, HepG2-NTCP cells were transduced with various AdVs, and tested for HBsAg and BIP/GRP78 levels by Western blot. Consistent with the qPCR data, BIP/GRP78 levels were significantly elevated in Ad-HBsAg-transduced cells (Figure 1C). AdV containing the 1.3× full-length HBV genome capable of HBV replication and production of all HBV proteins did not induce any of these UPR markers. To test the effect of HBV replication on these UPR genes, HepG2-NTCP cells were infected with high-titer infectious HBV generated in cell culture and gene expression was analyzed 1, 3, 7 and 10 days later. Despite a high HBV infection efficiency (Supplementary Figure S2), we did not detect significant changes in any of the PERK markers in these cells (Figure 1D).

### 3.2. Induction of PERK Pathway Genes Correlates with Level of HBsAg Expression

**Figure 2:**
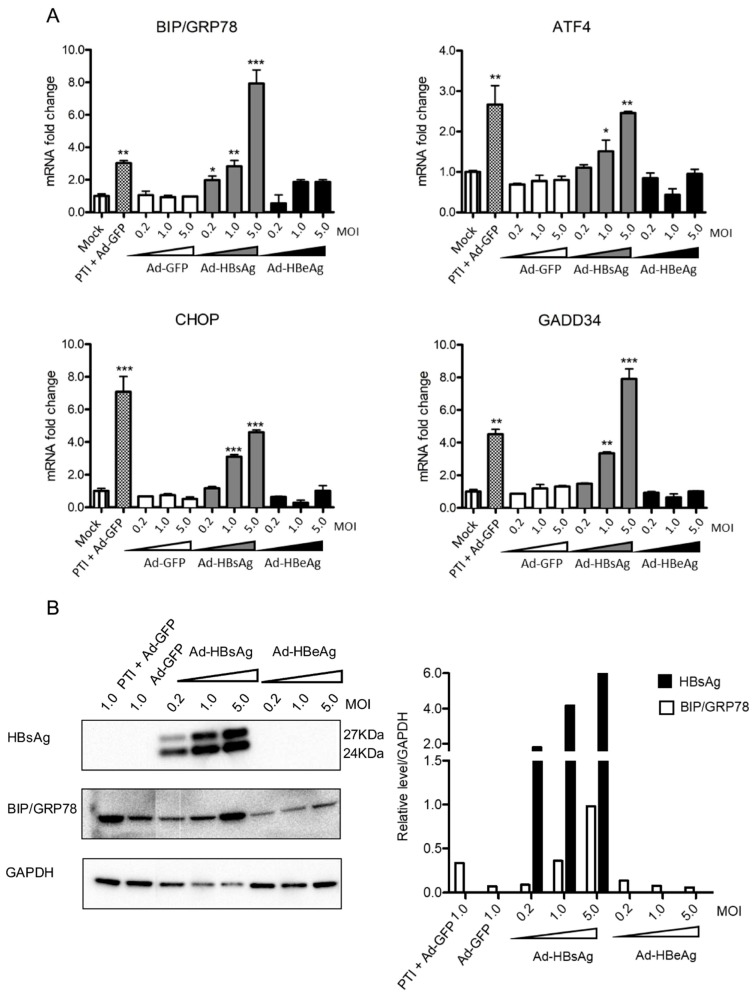
Dose-dependent induction of PERK pathway genes by HBsAg expression. Ad-GFP, Ad-HBsAg and Ad-HBeAg were transduced into HepG2-NTCP cells with increasing MOIs of 0.2, 1.0 and 5.0 in triplicate. Protein transportation inhibitor (PTI) was used as a positive control. (**A**) Three days after transduction, mRNAs of BIP/GRP78, ATF4, CHOP and GADD34 were determined by RT-qPCR. The Student’s unpaired two-tailed *t* test was applied for data analysis. *p < 0.05, **p < 0.01, ***p < 0.001. (**B**) Expression of HBsAg and ER stress marker BIP/GRP78 were measured by Western blotting (left panel) and signals quantified (right panel). All results were confirmed by three independent experiments.

Based on the above results, we reason that only the overexpression of SHBsAg, and not other HBV proteins mentioned, activates the PERK pathway. To examine this possibility and better ascertain the level of HBsAg expression necessary for PERK pathway activation, HepG2-NTCP cells were transduced with various MOIs (0.2, 1.0 and 5.0) of Ad-HBsAg. The PERK pathway markers were then tested by both qPCR and Western blot. As shown in Figure 2A, the mRNA levels of BIP/GRP78, ATF4, CHOP and GADD34 increased with increasing MOIs. On the contrary, no significant change was observed in the Ad-GFP or Ad-HBeAg transduced cells (Figure 2B). Western blot confirmed the qPCR data and showed increasing levels of BIP/GRP78 protein with higher MOIs of Ad-HBsAg. Our data suggest that a threshold of HBsAg expression may be necessary for the induction UPR. The level of HBsAg expression in Ad-HBsAg transduced cells is at least 20 times higher than that achieved in the HBV-infected cells, explaining the lack of UPR induction in HBV-infected cells (Supplementary Figure S3).

### 3.3. Activation of ER Stress Marker BIP/GRP78 by HBsAg at Single-cell Level

**Figure 3:**
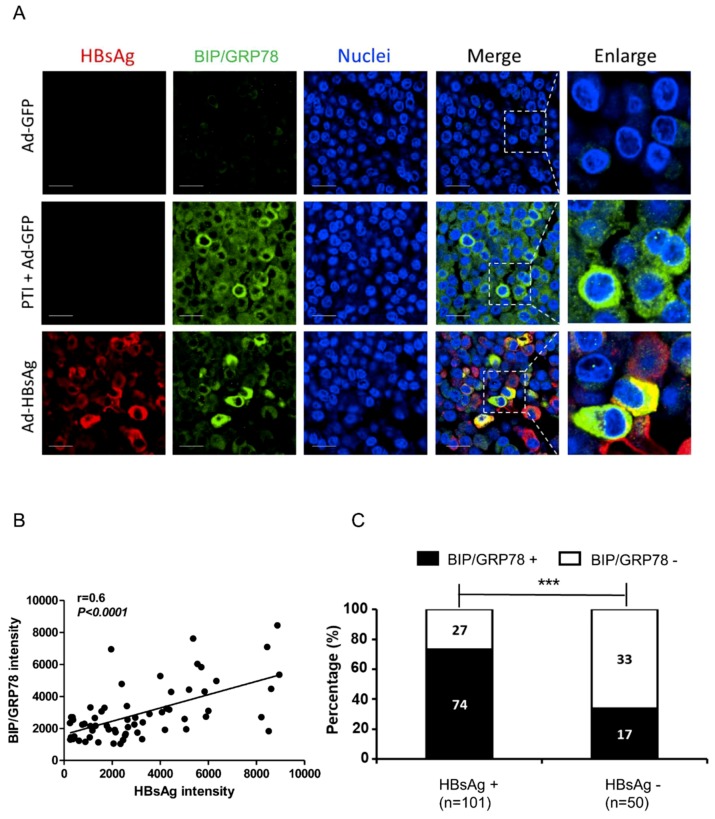
Co-localization of ER stress marker BIP/GRP78 and HBsAg. HepG2-NTCP cells were paraffin-embedded after transduction with Ad-GFP, Ad-HBsAg or treatment with PTI for six hours. (**A**) Immunofluorescent staining with anti-HBsAg and anti-BIP/GRP78 antibodies were performed three days after transduction. Nuclei were stained with Hoechst 33342. Scale bar represents 20 μm. (**B**) In Ad-HBsAg-transduced cells, the HBsAg and BIP/GRP78 intensities in each cell were measured, and their correlation was evaluated. (**C**) Ad-HBsAg transduced cells were grouped by HBsAg staining and analyzed for BIP/GRP78 signals. The ratio of BIP/GRP78 positive vs. negative cells was determined and shown. All results were confirmed by three independent experiments. *** *p* < 0.001.

To visualize ER stress at the cellular level, we performed immunostaining of ER stress marker BIP/GRP78 in AdVs-transduced cells. Three days after AdVs transduction or six hours after treatment with PTI, HepG2-NTCP cells were paraffin-embedded and mounted. The slides were then de-paraffinized, subjected to antigen retrieval procedure, and stained with antibodies against HBsAg and BIP/GRP78. Ad-GPF was used as a negative control and PTI + Ad-GFP as a positive control. The GFP in the Ad-GFP-transduced cells no longer fluoresces under the fixing/embedding condition for the antibody staining; thus the green represents BIP/GRP78 staining in recombinant adenovirus-transduced cells. As shown in Figure 3A, minimal BIP/GRP78 staining was detected in Ad-GFP transduced cells, but in PTI + Ad-GFP cells, BIP/GRP78 level was significantly elevated. In Ad-HBsAg transduced cells, BIP/GRP78 levels were substantially higher in cells with HBsAg expression, and colocalization of BIP/GRP78 and HBsAg was observed in many cells. 

To confirm the relationship between HBsAg and BIP/GRP78, a quantitative analysis was performed. A large number of Ad-HBsAg transduced cells were analyzed; the HBsAg and BIP/GRP78 intensities in each cell were measured and their correlation evaluated. As shown in Figure 3B, BIP/GRP78 expression has a positive correlation with HBsAg level (*r* = 0.6, *p* < 0.0001). In a separate analysis (Figure 3C), cells were divided into HBsAg-positive and -negative subgroups and BIP/GRP78 signals were measured accordingly. HBsAg-positive cells showed a significantly higher BIP/GRP78 positivity than HBsAg-negative cells [73.2% (74/101) vs. 34.0% (17/50), *p* < 0.0001].

### 3.4. Induction of Apoptosis in HepG2-NTCP Cells and PHHs by HBsAg Overexpression and ER Stress

**Figure 4:**
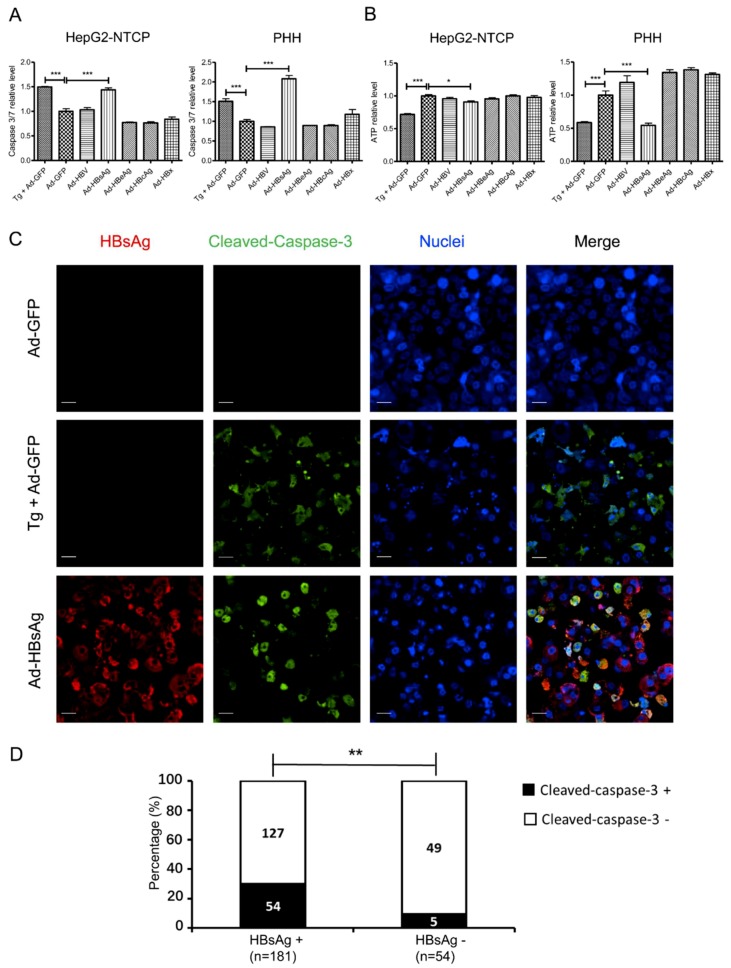
Induction of apoptosis by HBsAg overexpression. HepG2-NTCP cells and PHHs were transduced by AdVs with an MOI of 1 or treated with Thapsigargin (Tg, 1 µM for 24 h) as a positive control. (**A**) Five days after transduction, apoptosis was evaluated by the Caspase-3/7 activity assay. (**B**) Cell viability was measured by ATPlite. The Student’s unpaired two-tailed *t* test was applied for data analysis. *p < 0.05, ***p < 0.001. (**C**) In AdVs-transduced HepG2-NTCP cells, immunofluorescent staining for HBsAg (red) or cleaved-caspase-3 (green) was performed. Nuclei were stained with Hoechst 33342. Scale bar: 20 μm. (**D**) Ad-HBsAg transduced cells were grouped by HBsAg staining and measured for cleaved-caspase-3 signals. The ratio of cleaved-caspase-3 positive vs. negative cells was determined and shown. ** represents *p* < 0.01.

Under ER stress, UPR exhibits a dynamic and flexible network that responds to various inputs. On one hand, UPR can help the cells survive during ER stress, such as by expanding the ER membrane to enhance protein synthesis. On the other hand, when ER stress is prolonged and not resolved, the UPR can trigger apoptosis [24]. To determine the cell fate under prolonged ER stress caused by AdVs, we tested for evidence of apoptosis with the Caspase-Glo^®^ 3/7 system immunofluorescent assays five days after AdVs transduction. Cell viability was examined with the ATPlite assay. Cells were treated with Tg, a known apoptosis inducer, for 24 h as a positive control. Compared with Ad-GFP transduced cells, Caspase-3/7 activity was increased more than 1.5-fold in the positive control group and the Ad-HBsAg transduced cells. Using the ATPlite assay, the viability of Ad-HBsAg transduced cells was modestly lower, consistent with the gradual induction of apoptosis by prolonged ER stress (Figure 4A). Similar results were noted in primary human hepatocytes (Figure 4B). Staining for cleaved-caspase-3 was also performed as another marker of apoptosis. In HepG2-NTCP cells, those that were Ad-HBsAg transduced showed significantly increased cleaved-caspase-3 signals (Figure 4C). Further quantification was measured in Ad-HBsAg transduced cells (Figure 4D). Cells were divided into HBsAg-positive and -negative subgroups and cleaved-caspase-3 signals were measured accordingly. HBsAg-positive cells had significantly higher cleaved-caspase-3 positivity [29.8% (54/181) vs. 9.2% (5/54), *p* = 0.002]. However, some HBsAg-positive cells did not show cleaved-caspase-3 signals. It is possible that high HBsAg expression may be necessary but not sufficient to induce apoptosis in this experimental system.

### 3.5. ER Stress and Ground Glass Hepatocytes in HBV-Infected Liver

**Figure 5:**
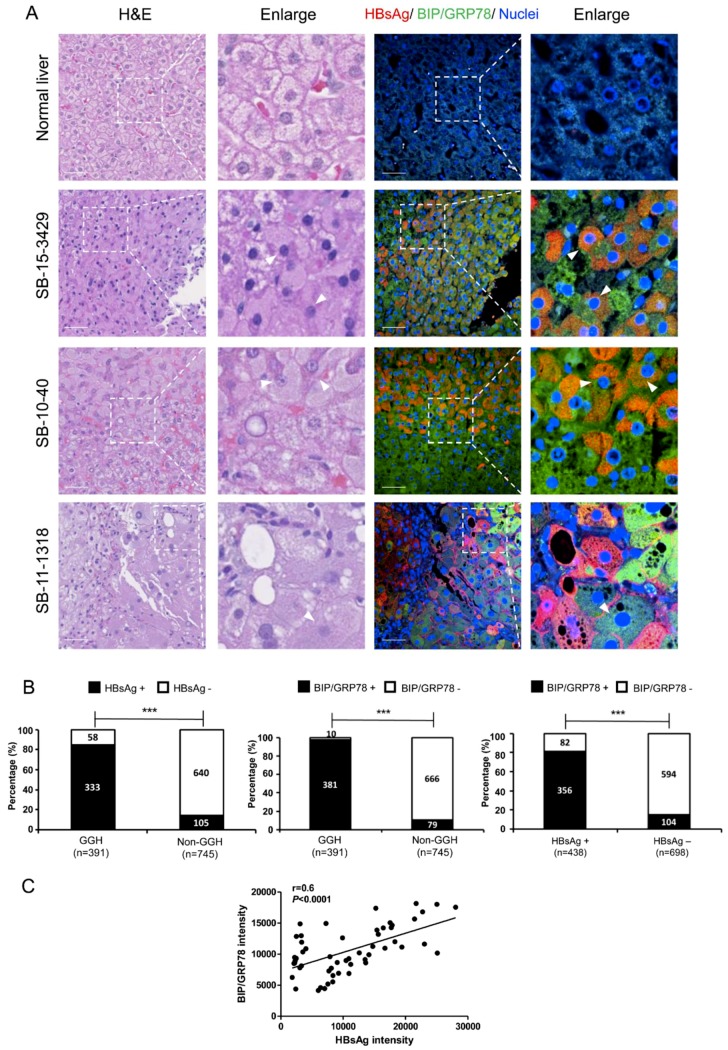
H&E and immunofluorescent staining of HBV-infected livers. (**A**) Paraffin-embedded liver specimens were evaluated by H&E and immunofluorescent staining. Staining of representative fields of normal liver and three HBV patients are shown. Areas with GGHs are magnified and marked (white arrowheads). HBsAg (red), BIP/GRP78 (green) and nuclei (blue) are shown. Scale bar represents 50 μm. (**B**) Quantifications were performed based on the staining of HBV patients shown above. The ratio of HBsAg-positive to negative cells (left) or BIP/GRP78 -positive vs. negative cells (middle) is shown. Similarly, groups based on HBsAg staining and the ratio of BIP/GRP78 -positive vs. negative cells were determined (right). *** *p* < 0.001. (**C**) In immunofluorescent staining on liver samples from chronic hepatitis B patients, the HBsAg and BIP/GRP78 intensities in each cell were measured, and their correlation was evaluated.

To delineate the relationship among HBV infection, ER stress and GGHs in vivo, we evaluated hematoxylin and eosin (H&E) and immunofluorescent staining on liver samples from chronic hepatitis B patients. Liver specimens of five healthy donators and seven HBV-infected patients were studied. The demographic and clinical information of the HBV patients is described in Supplementary Table S3. The representative fields of staining for three patients compared with a normal liver are shown in Figure 5A. Both HBsAg and BIP/GRP78 staining were strongly positive in HBV-infected livers. The distribution of HBsAg was predominately diffuse cytoplasmic (SB-15-3429, SB-10-40), though some membranous staining occurred (SB-11-1318). HBsAg-positive cells were strongly positive for BIP/GRP78 staining. Analyses of immunofluorescent and H&E staining showed that the majority of GGHs (>80%) were strongly positive for HBsAg and BIP/GRP78 as compared to the non-GGHs. Representative staining images of all seven patients are shown in Supplementary Figure S4.

To quantify the overall distributions of GGHs, HBsAg and BIP/GRP78 staining in HBV-infected livers (Figure 5B), we grouped hepatocytes based on GGHs vs. non-GGHs. 2–3 fields from the H&E staining were randomly selected from each patient and the corresponding fields of immunofluorescent staining for HBsAg or BIP/GRP78 were then shown. The numbers of HBsAg or BIP/GRP78-positive or -negative cells were counted in each group. The HBsAg and BIP/GRP78 staining were first compared between GGHs and non-GGHs. GGHs have significantly higher HBsAg and BIP/GRP78 positivity [HBsAg: 85.2% (333/391) vs. 14.1% (105/745), *p* < 0.0001; BIP/GRP78. 97.4% (381/391) vs. 10.6% (79/745), *p* < 0.0001]. Similarly, HBsAg-positive cells also showed substantially higher BIP/GRP78 staining comparing to HBsAg-negative cells [91.0% (356/391) vs. 14.0% (104/745), *p* < 0.0001] (Figure 5B). To further confirm the relationship between HBsAg and BIP/GRP78, a quantitative analysis was performed based on immunofluorescent staining. The HBsAg and BIP/GRP78 intensities in each cell were measured and their correlation evaluated. As shown in Figure 5C, BIP/GRP78 expression has a positive correlation with HBsAg level (*r* = 0.6, *p* < 0.0001).

Because prolonged and unresolved UPR can lead to apoptosis, we examined the GGHs for histological evidence of apoptosis. Based on a close examination of the H&E staining of these samples, our pathologist (DEK) did not identify any increased presence of acidophilic bodies (apoptotic hepatocytes) in association with the GGHs. Besides, no significant apoptosis signal was found in liver biospies of chronic hepatitis B patients through apoptosis marker staining (Supplementary Figure S5). 

## 4. Discussion

In this study, we aimed to define the role of SHBsAg overexpression in inducing ER stress and UPR. We demonstrated that overexpression of SHBsAg is associated with this pathological condition. In both HepG2-NTCP cells and PHHs, the PERK pathway of UPR was activated when SHBsAg was overexpressed. In HBV-infected liver biopsies, we showed that GGHs, which are marked by HBsAg accumulation within the ER lumen, display UPR with strongly positive BIP/GRP78 staining that co-localizes with HBsAg. 

We used two approaches to explore the interaction between HBV infection and UPR induction in vitro: an AdVs transduced system and an HBV infection system. Firstly, we evaluated the work efficiency of these systems. In addition to HBV genes, the GFP gene was inserted into the recombinant AdVs as an indicator of transduction efficiency in the former system. As shown in Supplementary Figure S1, GFP was observed in all AdVs-transduced cells and indicated similar transduction efficiency. HBsAg and HBeAg ELISAs were performed to verify their expression. Additionally, HBV DNA was measured by qPCR after AdVs transduction. Though HBx was not measured in our research due to the lack of an effective measurement protocol, this system has been widely used and HBx expression verified in previous studies [17,18]. In the latter system, we use immunofluorescent staining to test the infection efficiency. As shown in Supplementary Figure S2, more than 80% of cells were HBsAg positive, suggesting this in vitro system supports HBV infection efficiently. 

Next, we identified UPR under the different systems. In the AdVs transduced system, as shown in Figure 1, PERK pathway was activated in Ad-HBsAg transduced cells, indicating that the overexpression of HBsAg induces UPR. Because the same phenomenon was not observed in Ad-HBV transduced HepG2-NTCP cells, we speculate that a threshold of HBsAg expression level may be necessary for the induction of UPR. As shown in Supplementary Figure S3, the level of HBsAg expression in Ad-HBsAg transduced cells was at least 10 times higher than that achieved in the Ad-HBV transduced cells. This could be because Ad-HBV transduced cells are responsible for HBV replication as well as the production of all HBV proteins. In the HBV infection system, evidence of the induction of UPR was similarly absent. We reasoned that, such as in the Ad-HBV transduced cells, the expression of HBsAg was too low to activate UPR (Supplementary Figure S3). Interestingly, Ad-HBeAg was found to activate UPR in PHHs. This may be explained by the higher sensitivity of PHHs as compared to HepG2-NTCP cells. It has been reported that the sensitivity of PHHs in response to drug hepatotoxicity is more than 25% higher than HepG2-NTCP cells [25]. As a hepatocarcinoma cell line, HepG2-NTCP cells may be more tolerant to cancer-related pathways such as UPR. The potential oncogenic nature of HBeAg should be further explored.

Previous studies have reported two types of GGHs associated with HBV infection [4]. Type I GGHs are scattered, single cells that have been reported to harbor HBV mutants with pre-S1 region deletion. Type II GGHs are clusters of cells and are more often correlated with active HBV disease. They were thought to harbor HBV mutants with pre-S2 region deletions [4,26]. Mutant pre-S proteins were thought to block the secretion of HBsAg, consequently causing intracellular accumulation of HBsAg and resulting in liver injury. Work in the transgenic mouse model supported the pathogenic role of pre-S1 protein when it is overexpressed [27]. While pre-S deletion mutants were found in GGHs, wild-type HBV still constitutes a great majority of clones in the same sample [4]. Whether these pre-S mutants in the presence of abundant wild-type HBsAg can indeed cause massive accumulation of intracellular HBsAg remains unclear. It is also possible that the integrated HBV sequences in HBV-infected hepatocytes can direct non-physiological expression of HBsAg, leading to ER stress, UPR and formation of GGHs. 

The alternative explanation is that the precursor cells of these GGHs may be functionally deficient in the proper assembly and secretion of HBsAg, resulting in a substantial accumulation of intracellular HBsAg. Our study is consistent with the notion that overexpression of HBsAg can overwhelm the cellular machinery for HBsAg assembly/secretion and result in UPR. In cell culture, HBV infection is adequately handled by normal cellular machinery and thus does not typically cause abnormal accumulation of HBsAg and thus UPR. In chronic HBV infection in vivo, various pathological mechanisms as a result of chronic inflammation may cause unduly stress on the hepatocytes and thus a deranged cellular process leading to defective HBsAg assembly and processing. Due to a lack of HBsAg sequence information in our data, the relative contribution of either mechanism, one attributed to viral factors and the other to pathological host response or a combination of both, needs to be further defined.

Upon ER stress, cells activate the UPR to reduce misfolded or unfolded protein load through multiple adaptive mechanisms in order to maintain homeostasis [23,24]. First, through the extension of ER membrane, the ER capability increases to help deal with abnormal proteins. Second, by blocking key protein synthesis, an adaption can be achieved transiently. Third, intracellular proteolytic pathways are activated to reduce the overall protein load. However, if the ER stress is prolonged and unresolved, the UPR can lead to activation of downstream factors such as CHOP, Bcl-2, Caspase-3, -7, etc., and trigger apoptosis [23,28,29]. This adaptive process is a physiological response to cell injury as the body tries to eliminate injured and dysfunctional cells. In our study, at a certain threshold of HBsAg expression, the UPR is induced with BIP/GRP78 induction. Three days after HBsAg overexpression, CHOP, which is an early apoptosis marker involved in ER stress [30], was significantly elevated. At five days, Capase-3/7 increased three-fold as a sign of ER stress-induced apoptosis progressing from the commitment phase to the execution phase [31].

Previous studies have reported on the interaction of HBV and apoptosis, and the results are controversial. HBV or HBX has been shown to inhibit cellular apoptosis, thereby facilitating viral proliferation and promoting HCC progression [32,33,34]. On the other hand, induction of apoptosis by HBV has also been reported by others [35,36,37]. Many of the previous studies were not performed in infectious cell culture systems nor validated by in vivo models, thus raising concerns about the biological relevance of those findings. During the natural infection process, HBV may interact with various apoptotic pathways to support its productive infection, but the degree of interaction probably does not reach the threshold of apoptosis or other cell death pathways, the regulation of which is quite complex. As mentioned above, during chronic HBV infection, the normal HBV replicative process may be disturbed by dysregulation of certain cellular process or accumulation of viral mutations in some infected cells, resulting in an aberrant activation of these pathways and leading to apoptosis. These cells, while undergoing prolonged ER stress, may adapt via either mutational events or epigenetic modifications to overcome the apoptotic signaling and become transformed. The GGHs, as illustrated in our study, may represent such a transition point in chronic hepatitis B. 

In GGHs, we noted abundant evidence of ER stress but did not observe any overt apoptosis. It is possible that many precursor cells of GGHs may undergo apoptosis because of the prolonged and unresolved ER stress. However, some of these cells may adapt to chronic ER stress, survive, become GGHs and progress to cancerous cells. Sustained activation of ER stress endows malignant cells with more tumorigenic capacity. Many cancers arise in the context of chronic inflammation and cellular stress, including ER stress [38]. Recent studies reported that ER stress response can hinder anti-tumor immunity by interfering with immune cells in the tumor microenvironment [38,39,40].

Other premalignant pathways have been reported to be associated with the formation of GGHs. Expression of vascular endothelial growth factor-A (VEGF-A) is significantly elevated in GGHs [41]. VEGF-A is an important regulator of normal and tumor angiogenesis. VEGF-A can stimulate the proliferation of cancer cells by acting as an autocrine growth factor [42]. Activation of mammalian target rapamycin (mTOR) has also been observed in GGHs and HBV-associated HCC [43]. Activated AKT/mTOR signaling pathway has been reported in many types of human cancers such as liver and colon cancers. [41,42,44]. 

Chronic HBV infection is etiologically linked to the development of HCC, which has the fastest increasing death rate from cancer in the world [45]. It is tempting to speculate that the GGHs may be the precursor to cancerous cells by undergoing additional genetic and epigenetic alternations. Interestingly, GGHs have been reported as a marker of HCC in chronic hepatitis B [46,47,48]. In this study, we demonstrate the involvement of the HBsAg in the activation of ER stress and downstream pathways. Further characterization of these mechanisms should provide valuable insight into the pathogenesis of GGHs and HBV-associated HCC development. 

## Supplementary Materials

The following are available online at https://www.mdpi.com/1999-4915/11/4/386/s1, Figure S1: Expression of HBV genes by recombinant AdVs in HepG2-NTCP, Figure S2: HBV infection in HepG2-NTCP cells, Figure S3: Expression of HBsAg in HepG2-NTCP cells, Figure S4: Immunofluorescent staining for HBV and UPR marker in HBV-infected liver samples, Figure S5: Immunofluorescent staining for apoptotic marker in HBV-infected liver samples, Table S1: Sequences of the primers for quantitative real-time PCR, Table S2: Information of antibodies, Table S3: Characteristics of chronic hepatitis B patients.
