# Supplementary material for: Hepatitis B Surface Antigen Activates Unfolded Protein Response in Forming Ground Glass Hepatocytes of Chronic Hepatitis B
Source: Viruses. 2019 Apr 25;11(4):386. doi: 10.3390/v11040386 (PMC6520809; doi:10.3390/v11040386)
Supplement: Supplementary file 1 [file viruses-11-00386-s001.pdf]

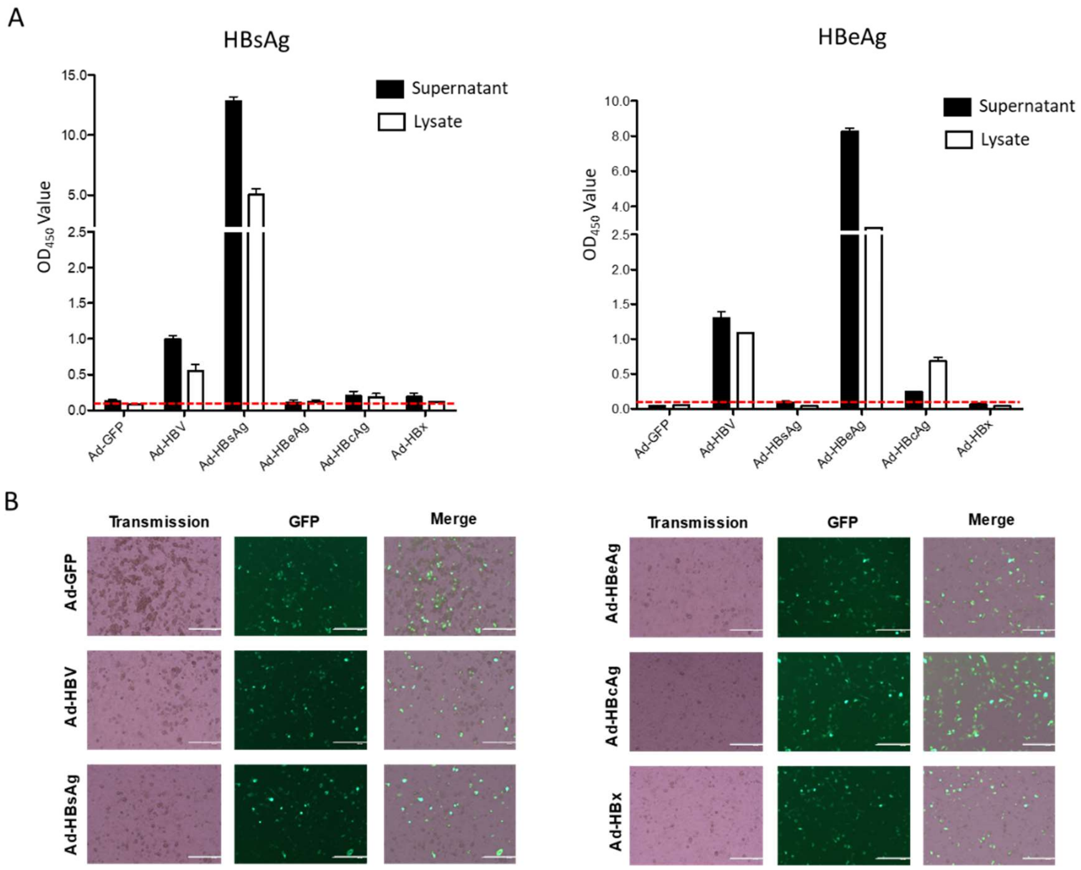

Fig. S1. Expression of HBV genes by recombinant AdVs in HepG2-NTCP. HepG2-NTCP cells were transduced with recombinant AdV expressing various HBV genes at a MOI of 1. **(A)** HBsAg and HBeAg in both supernatant and cell lysate were measured by ELISA. Red-dotted line shows the cut-off value (0.1). **(B)** GFP was visualized by fluorescent microscope. Scale bar represent 100 µm.

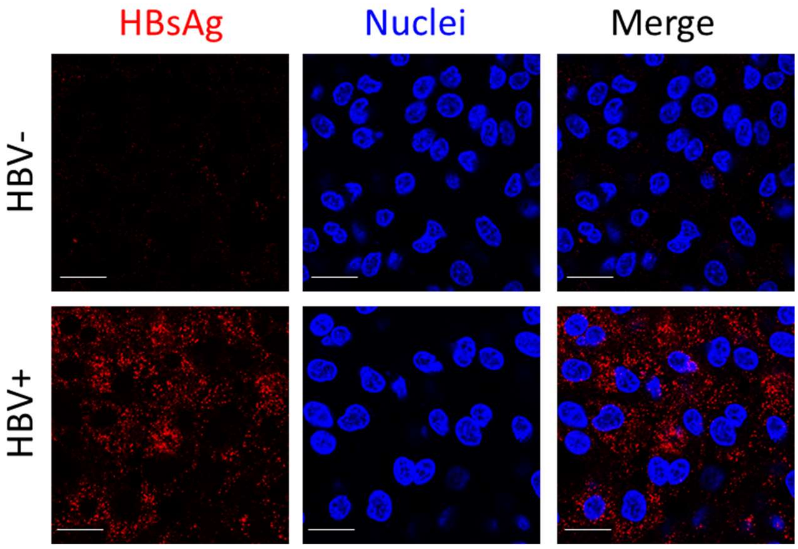

Fig. S2. HBV infection in HepG2-NTCP cells. HepG2-NTCP cells were infected with or without infectious HBV at a MOI of 300. Eight days after infection, immunofluorescent staining with anti-HBs antibodies were performed. Nuclei were stained with Hoechst 33342. Scale bar represents 20 μm.

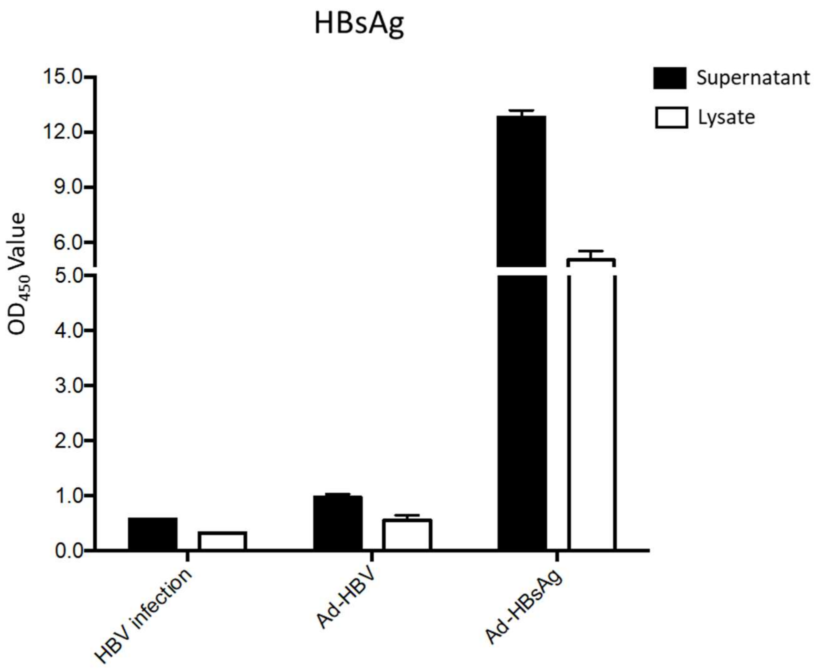

Fig. S3. Expression of HBsAg in HepG2-NTCP cells. HepG2-NTCP cells were transduced with Ad-HBV and Ad-HBsAg (MOI of 1). HepG2-NTCP cells were infected with infectious HBV at a MOI of 300. HBsAg levels in the supernatant and cell lysate were measured by ELISA on day three. Samples from Ad-HBsAg were diluted five-fold before testing, and the resulting OD_{450} values were multiplied by five in comparison to other samples.

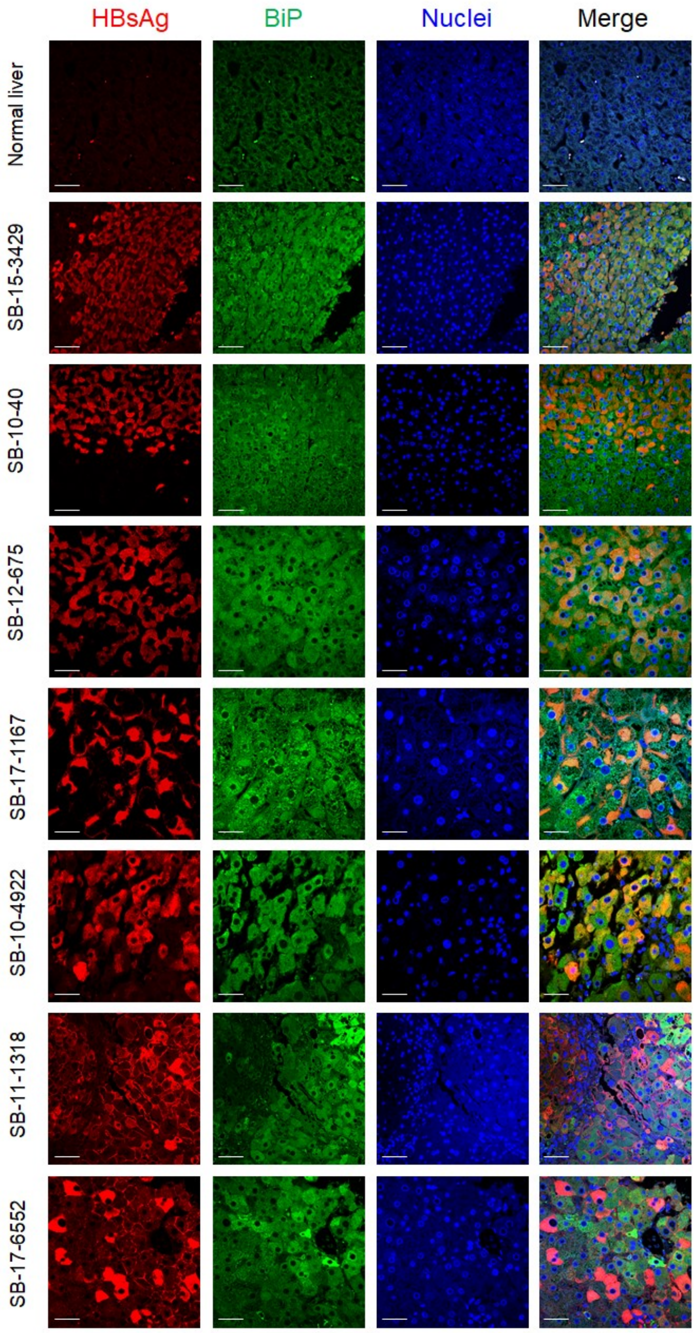

Fig. S4. Immunofluorescent staining for HBV and UPR marker in HBV-infected liver samples. Paraffin-embedded liver specimens from healthy donators and HBV-infected patients were enrolled for immunofluorescent analysis. HBsAg (red) and BiP (green) are shown. Nuclei were stained with Hoechst 33342. All five liver samples from health controls have the same staining pattern and thus only one is shown here. Representative staining fields of specimens from seven HBV patients are shown. HBsAg staining were cytoplasmic-diffuse in first five patients (SB-15-3429, SB-10-40, SB-12-675, SB17-1167, SB-10-4922) and membranous in last two patients (SB-11-1318, SB-17-6552). Scale bar represents 50 μm.

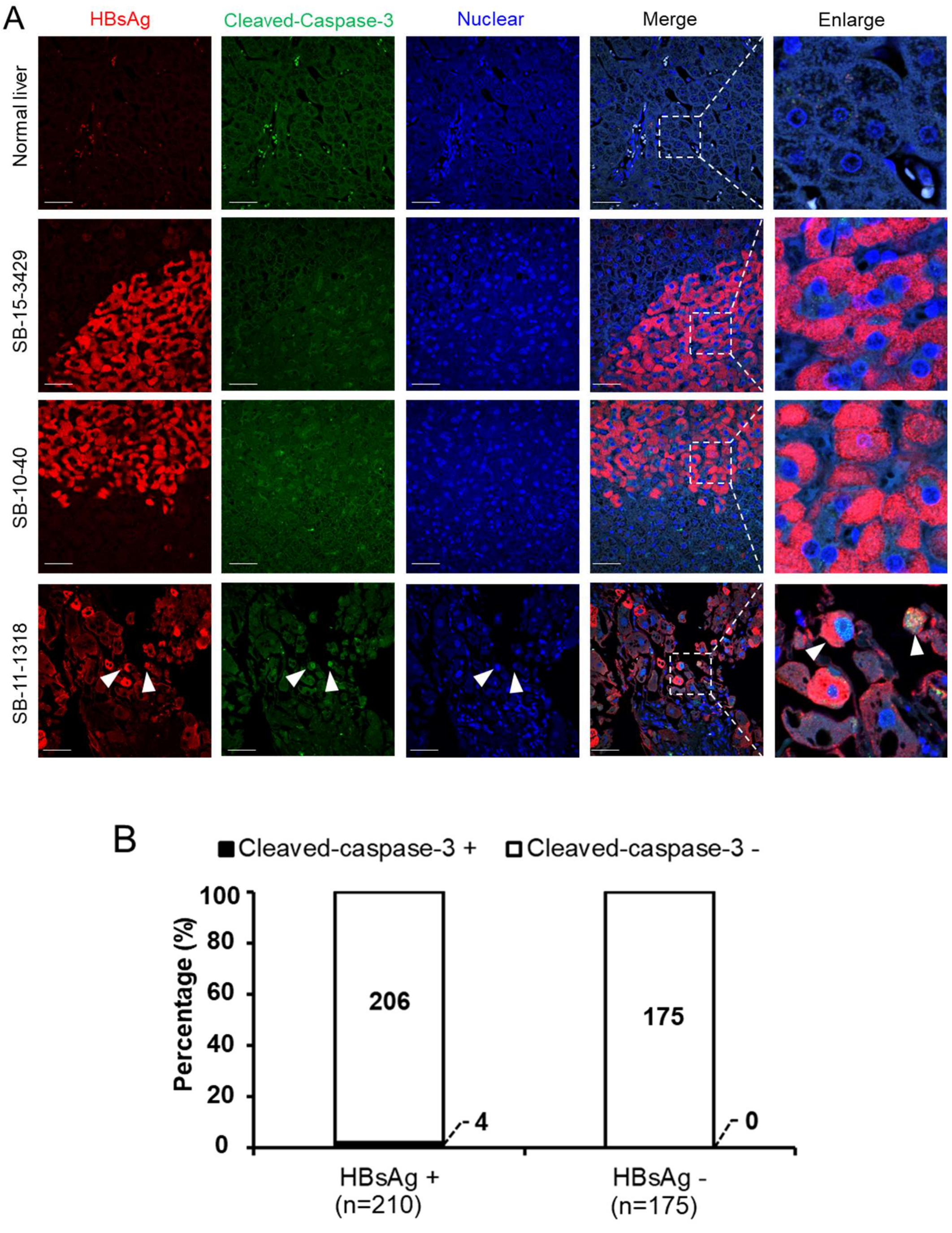

Fig. S5. Immunofluorescent staining for apoptotic marker in HBV-infected liver samples. (A) Paraffin-embedded liver specimens from health donors and HBV-infected patients are stained for apoptotic marker (cleaved-caspase-3). Representative normal liver and HBV-infected livers (SB-15-3429, SB-10-40 and SB-11-1318) are shown. HBsAg (red) and cleaved-caspase-3 (green) are

shown. Nuclei are stained with Hoechst 33342. White arrowhead indicates apoptotic hepatocytes. Scale bar represents 50 µm. **(B)** Quantification is performed based on the staining of HBsAg. The ratio of cleaved-caspase-3-positive to negative cells is shown.

Table S1 Sequences of the primers for quantitative real-time PCR

Target Gene	Forward primers	Revered primers
TBP	5'-TATAATCCCAAGCGGTTTGC-3'	5'-CTGTTCTTCACTCTTGGCTCCT-3'
GRP78	5'-CTGGGTACATTTGATCTGACTGG-3'	5'-GCATCCTGGTGGCTTTCCAGCCATTC-3'
ATF4	5'-GTTCTCCAGCGACAAGGCTA-3'	5'-ATCCTGCTTGCTGTTGTTGG-3'
CHOP	5'-AGAACCAGGAAACGGAAACAGA-3'	5'-TCTCCTTCATGCGCTGCTTT-3'
GADD34	5'-CGCCCAGAAACCCCTACTCAT-3'	5'-TCGGAGAAGCGCACCTTTCT-3'

Table S2 Information of antibodies

Target Antigen	Clone	Catalog No.	Supplier	Assay/Concentration
GAPDH	6C5	SC-32233	Santa Cruz Biotechnology	WB:1:300
BIP/GRP78	C50B12	3177	Cell Signaling Technology	WB: 1:1000;
				IF: 1: 200~500
HBsAg	2945	DMABT-51328MH	Creative-Diagnostics	WB: 1:200; IF: 1:100
Cleaved Caspase-3	Asp175	9661	Cell Signaling Technology	IF: 1:200~400

WB: Western Blotting; IF: Immunofluorescence

Table S3 Characteristics of chronic hepatitis B patients

Case No.	Age	Gender	HBV DNA (IU/mL)	HBeAg	ALT (U/L)	AST (U/L)	Ishak
SB-15-3429	23	M	<20	-	34	26	0
SB-10-40	28	M	3.43×10^6	+	77	48	2
SB-12-675	27	M	ND	-	74	35	0
SB-17-1167	48	M	3.40×10^5	-	73	47	3
SB-10-4922	43	M	ND	-	49	20	1
SB-11-1318	32	M	5.53×10^7	+	93	47	5
SB-17-6552	25	M	1.17×10^8	+	40	20	0

ALT: alanine aminotransferase; AST: aspartate aminotransferase; M: male; ND: not detected.
